# Studying the impact of chitosan salicylaldehyde/schiff base/CuFe_2_O_4_ in PC3 cells via theoretical studies and inhibition of PI3K/AKT/mTOR signalling

**DOI:** 10.1038/s41598-025-86096-7

**Published:** 2025-02-03

**Authors:** Ghada H. Elsayed, Asmaa M. Fahim

**Affiliations:** 1 Hormones Department, Medical Research and Clinical Studies Institute, and Stem Cell Lab, Centre of Excellence for Advanced Sciences, National Research Centre, Dokki, Cairo, 12622 Egypt; 2 Department of Green Chemistry, National Research Centre, Dokki, Cairo, 12622 Egypt

**Keywords:** Chitosan derivatives, Prostate cancer, Cytotoxicity, PI3K/AKT/mTOR, Theoretical studies, Biochemistry, Cell biology, Chemistry

## Abstract

In this elucidation, the nucleophilic attack of salicyladehyde with chitosan, which was obtained from the shrimp shell, afforded the cellulose aldehyde (Schiff base), and then the dispersion of CuFe_2_O_4_ on the surface of cellulose aldehyde gave the novel nanomaterial of bimetallic oxide, which was confirmed through spectral analysis such as FT-IR, NMR, SEM, and XRD analysis. Moreover, the anti-proliferative effect of chitosan, chitosan salicylaldehyde, and chitosan salicylaldehyde/CuFe_2_O_4_ was evaluated in **PC3** human prostate cancer cells and **HSF** normal human skin fibroblasts. After 48 h, **PC3** cell proliferation was significantly inhibited by chitosan salicylaldehyde/CuFe_2_O_4_ and chitosan salicylaldehyde (IC_50_ = 35.3 and 45.55 µg/ml, respectively) without any effects on normal **HSF** cells. The mRNA expression levels of PI3K, AKT, mTOR, and CCND1 were examined in **PC3**-treated cells by using QRT-PCR, and the results demonstrated that, by down-regulating the expression levels of these genes, chitosan salicylaldehyde/CuFe_2_O_4_ significantly affected prostate cancer cell proliferation, progression, and autophagy more than chitosan salicylaldehyde. Furthermore, the docking stimulation of the chitosan derivatives with different proteins showed the presence of CuFe_2_O_4_ particles effect on the interaction inside their pockets and increased the activities, and it’s related to biological evaluation. Additionally, the theoretical investigation of these chitosan derivatives and the determination of their physical descriptors showed the activity of bimetallic oxide and the presence of electrostatic hydrogen bond interaction. Finally, these findings may suggest that chitosan salicylaldehyde/CuFe_2_O_4_ has a promising anticancer impact against prostate cancer.

**Supplementary Information:**

The online version contains supplementary material available at 10.1038/s41598-025-86096-7.

## Introduction

Prostate cancer (PC) is the second most often diagnosed tumor and the sixth most common cause of cancer-related mortality in males, with 1,414,259 new cases and 375,304 deaths recorded globally in 2020^1,2^The incidence rates and death have a period of stability, following a significant drop associated with prostate-specific antigen testing^3^. Age and ethnicity are the primary risk variables, but diet and family history are also significant factors^4,5^. Prostate cancer germline mutations are extremely rare and mostly impact the genes related to the DNA damage response^6^. Chitosan, a naturally occurring polysaccharide, is present in various seafood varieties, including crayfish, prawns, and crabs, with a higher concentration in their shells. The main characteristics of chitosan are its non-toxicity, biodegradability, and biocompatibility^7,8^. Chitosan exhibits greater solubility in acidic environments because of the protonation of amino groups, which occurs throughout the chitosan backbone and contributes to its high concentration of reactive free protonable amino groups. These groups can be chemically altered via a range of chemical processes to improve the material’s solubility, biocompatibility, and targeting efficacy^9,10^. As a result, chitosan may find use in several domains^11^. Certain medicinal compounds conjugated with derivatives of chitin or chitosan have demonstrated remarkable anticancer efficacy with fewer side effects than the original medications^12^. The PI3K-AKT pathway is an essential kinase signaling network in the development of cancer. The aberrant activation of the PI3K-AKT signalling pathway has been linked to many cancers, including endometrial, hepatic, breast, colorectal, prostate, and cervical cancers^13^. Thus regulation and blockage of this kinase and its essential ligands might be a possible method in cancer therapy. According to reports, chitosan and its derivatives inhibit AKT phosphorylation in a dose-dependent manner and are utilized to stop AKT activity in several cancer types^14^. It is noteworthy that the PI3K-AKT pathway stimulates the G1/S cell cycle in addition to inhibiting apoptosis. It is well known that to stop cyclin D1 breakdown, AKT is known to phosphorylate and inhibit GSK3β^15^Cyclin D1 (CCND1), a critical modulator of cell cycle development, is the primary cyclin involved in the transition of cells from the G1 to S phase and also is essential for the pathogenesis of cancer^16^. In several different types of cancer, CCND1 is overexpressed. Numerous mutations in CCND1 have been linked to the incidence, progression, prognosis, and management of malignancies^17–19^. According to several studies, the PI3K-Akt-mTOR pathway may be a significant factor in prostate cancer as a therapeutic target and/or a predictive biomarker for the development, and behavior of the illness^19^. Oncogenic activation of the phosphatidylinositol-3-kinase (PI3K), protein kinase B (PKB-AKT), and mammalian target of rapamycin (mTOR) pathway is a frequent event in prostate cancer that facilitates tumor formation, disease progression and therapeutic resistance. Recent findings suggest that the intricate interactions between the PI3K-AKT-mTOR pathway and many associated cell signalling cascades might further accelerate prostate cancer growth^20,21^.

The aim of this study is the examination of the interaction of the bimetallic oxide of chitosan salicylaldehyde which was obtained from the nucleophilic attack of chitosan with salicylaldehyde and its formation of the chitosan-salicylaldehyde Schiff base, which can be adsorbed with CuFe_2_O_4_ nanomaterials and afforded the corresponding chitosan/salicylaldehyde/CuFe_2_O_4_ and these compounds were confirmed through FT-IR, NMR, SEM, and XRD. Furthermore, we will examine how these chitosan derivatives affect the proliferation of **PC3** cancer cells and **HSF** normal cells. We also assessed the expression of the genes PI3K, AKT, mTOR, and CCND1 in **PC3**-treated cells. Also, the docking simulation of these chitosan derivatives confirmed the activities of both the Schiff base and its presence of metallic oxide. Moreover, the computational investigation of these chitosan derivatives with DFT/B3LYP/LAN2DZ(G) basis set showed its HOMO-LUMO gap energy and its physical descriptor which confirmed the presence of metal oxide increase the activity due to the electrostatic hydrogen bond interaction.

## Experimental section

### Instruments and techniques

Gallenkamp melting point apparatus was used for measuring melting points. Moreover, the Shimadzu FT-IR 8101 PC infrared spectrophotometer recorded the IR spectra. The ^1^HNMR was determined in DMSO-d_6_ at 300 MHz on a Varian Mercury VX 300 NMR spectrometer (^1^H at 300 MHz, ^13^C at 75 MHz) exhausting trimethyl silane as an internal typical. Scanning electron microscopes (SEM) were investigated utilizing JEOL JXA-840 A electron probe Micro analyzer Company and were air-dried before imaging, and images were obtained using an accelerating voltage of 10–15 kV.

### Chemicals and reagents

Salicylaldehyde and all chemicals were ordered from Sigma Aldrich Company.

### Hydrolysis of shrimp shell with 60%NaOH

The shrimp shell was washed several times and dried in an oven at 150^o^C for days soaked in a solution of 60% NaOH and refluxed for 5 h which turned on demineralization, deprotonation, and then finally deacylated and then cooled, and turned to a brown color filtered and washed several times with EtOH/H_2_O for purification^22^.

### Reaction of chitosan with salicylaldehyde

The reaction of chitosan(1 g,3mmol) in 100 ml of 10% acetic acid solution to obtain the homogenous solution in 1 h, added the salicylaldehyde(3 ml,3mmol) was added dropwise of chitosan solution and then after another 1 h to get the yellow gel of chitosan-salicylaldehyde Schiff base (**3**)^23,24^.

### Preparation of CuFe_2_O_4_ nanoparticles

SONOPULS HD 2070 - Ultrasonic homogenizer - max. 70 W was used to mix a mixture of CuCl_2_⋅2H_2_O (0.5 g) and FeCl_3_⋅6H_2_O (1.5 g) solutions in 30 ml deionized H_2_O and add 1 M NaOH (25 ml). The solid bimetallic precipitate was then repeatedly washed with H_2_O and EtOH and dried over 80^o^C for 12 h^25^.

### Adsorption of chitosan-salicylaldehyde Schiff base(3) with CuFe_2_O_4_

Furthermore, the formed chitosan-salicylaldehyde Schiff base (**3)** (1 g) in 25 ml distilled water was stirred with the solution of CuFe_2_O_4_(0.25 g) for 5 h to get the chitosan-salicylaldehyde/CuFe_2_O_4_ (**4**), to form nanoparticles of CuFe_2_O_4_ to afford C_29_H_38_CuFe_2_N_2_O_13_ (707), (pale brown) with 88%yield, (**4**)

### Cell lines and culture conditions

The **PC3** human prostate cancer cells and **HSF** human skin fibroblasts were procured from the American Type Culture Collection (ATCC) and cultured under appropriate conditions. In RPMI and DMEM medium supplemented with 10% fetal bovine serum (FBS), 100 U/ml penicillin, and 100 µg/ml streptomycin sulfate, **PC3**, and **HSF** cells, respectively, were cultured at 37 °C in a humidified 5% CO_2_. The cells were harvested after trypsinization (0.025% Trypsin and 0.02% EDTA). When the cell density reached approximately 80%, cells were split for further culture. 

### Anti-proliferative assay

Anti-proliferative effect was estimated using a neutral red uptake assay according to Repetto et al.^26^. The neutral red uptake assay provides a quantitative estimation of the number of viable cells in a culture, and it is based on the ability of viable cells to incorporate and bind the supravital dye neutral red in the lysosomes. In a 96-well plate, **PC3** cancer cells and **HSF** normal cells (10^4^ cells/well) were incubated for 48 h with different concentrations of chitosan derivatives (25, 50, 100, and 200 µg/ml), untreated cells (negative control), and doxorubicin (Dox, Mr = 543.5), the positive control. A neutral red working solution (0.4 µg/ml) (Sigma-Aldrich) was incubated overnight at 37 °C in the same manner as the treated cells. In each well of the incubated cells, culture media were removed and a neutral red medium (100 µl) was added and then incubated for 2 h to allow for vital dye incorporation into living cells. The neutral red media were decanted, and Dulbecco’s PBS buffer (150 µl) was added to each well. Dye was extracted from the cells by adding extraction buffer [150 µl, 1% acetic acid: 50% ethanol (96%): 49% deionized H_2_O], followed by rapid agitation for at least 10 min on a micrometer plate shaker. The extract’s neutral red color intensity was measured at 450 and 630 nm as excitation and emission wavelengths in a microtiter plate reader spectrophotometer (Sorin, Biomedica S.p.A., Milan, Italy). The IC_50_ of the tested compounds were calculated using the relation between the used log concentrations and the neutral red intensity value. The examined chitosan derivatives were dissolved in dimethyl sulfoxide (DMSO), and the final concentration in the cells was less than 0.2%. Mean data was utilized for calculations, and each experiment was run in triplicate. The following formula was used to determine the percentage of cell viability: 

$$\% Cell \, viability=(absorbance \, of\: compounds \:treated \:cells/absorbance \:of\: control\: cells) \times 100.$$

### QRT-PCR analysis

Using an RNAeasy mini kit (Qiagen, Germany), RNA was isolated from **PC3** prostate cancer cells (3 × 10^4^ cells/well) that had been treated for 48 h. Then, using a NanoDrop one microvolume UV spectrophotometer (Thermo Fisher Scientific, USA), the concentration and purity of the extracted RNA were determined. The Revert Aid First Strand cDNA Synthesis Kit (Thermo Fisher Scientific, USA) was used to convert RNA from each treatment to first-strand cDNA in compliance with the manufacturer’s instructions. The expression levels of PI3K, AKT, mTOR, and CCND1 genes were normalized about the β-Actin transcript and quantified using the 2^−ΔΔCT^method^27^, using Maxima SYBR Green qPCR Master Mix (2X) (Thermo Fisher Scientific, USA). The reaction conditions were as follows: 95 °C for 10 min, 95 °C for 15 s, 60 °C for 30 s, and 72 °C for 30 s with a total of 40 cycles of amplification. The estimation of gene expression was performed using the DNA Technology Detecting Thermocycler DT Lite 4S1 (Russia). Primer sequences are included in Table 1.

**Table 1:** Primer sequences of target genes.

Gene	Primer forward (5’−3’)	Primer reverse (5’−3’)	References
**β-Actin**	CCTTCCTGGGCATGGAGTCCT	GGAGCAATGATCTTGATCTTC	^28^
**PI3K**	GAACGAGTGGTTGGGCAATG	CCTCGCAACAGGTTTTCAGC	^29^
**AKT**	TCTATGGCGCTGAGATTGTG	CTTAATGTGCCCGTCCTTGT	^30^
**mTOR**	GCTTGATTTGGTTCCCAGGACAGT	GTGCTGAGTTTGCTGTACCCATGT	^29^
**CCND1**	GAGGAAGAGGAGGAGGAGGA	TAACGTTGAGGGGCATCG	^28^

### Statistical evaluation

For the data, a mean ± standard error mean is shown. The data are determined using Sigma plot version 11. Significant differences between the investigated chitosan derivatives were found by using the student t-test to analyze the data. A statistical significance level of *P* < 0.05 was employed. A data set may be repeated more than once.

### Docking investigation

Molecular docking studies were conducted for the newly developed heterocyclic. We used standard bond lengths and angles within the MOE program, and the analyses were carried out using Discovery Studio Client (version 4.2)^31,32^. After optimizing the geometry, we thoroughly explored various conformations systematically until reaching an RMS gradient of 0.01 Å. The resulting conformations underwent energy minimization using the Confirmation Examination module within Auto Dock Vina. This process was based on the Structure of Crystal structure of Focal Adhesion Kinase (FAK)(PDBID:1mp8)^33^, PH domain of AKT-like kinase in Trypanosoma cruzi(PDBID:8ozz)^34^, DEPTOR DEP domain tandem (DEPt)(PDBID:7ped)^35^, Crystal structure of CDK4 in complex with CyclinD1 and P27(PDBID: 6p8e)^36^, Structure determinants of phosphoinositide 3-kinase inhibition by wortmannin, LY294002, quercetin, myricetin and staurosporine (PDBID:1e7u)^37^ We conducted nine separate docking simulations using default parameters, and the conformations were selected based on the alignment with overall statistics, E conformation, and compatibility with the pertinent amino acids in the respective protein’s binding pocket.

### Theoretical analysis

#### Computational investigation

Geometrical optimization using DFT/ B3PW91/LANDZ2 level of theory for metals utilizing the Berny technique^22,23^was achieved through the Gaussian 09 W program^24–26^. The HOMO-LUMO energy gap was investigated and viewed through the Gauss-View program^27,28,38^.

## Results and discussion

### Chemistry section

**Fig. 1:**
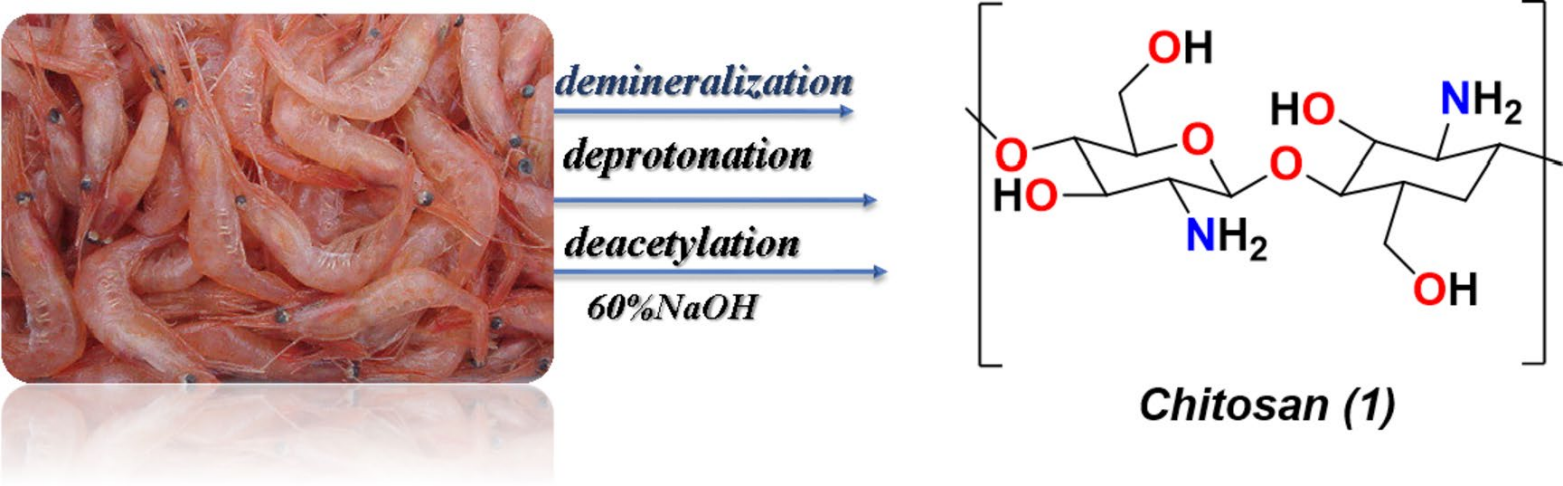
Process of extraction of chitosan from shrimp shell

The waste minimization of different sources to obtain the starting material such as shrimp shell which is the source of chitosan we take the shrimp shell and washed it several times and then soaked it in 60%NaOH and refluxed for 5 h then obtained the chitosan compound (1) after different process such as Fig. 1 which is began with demineralization (Chitin), deprotonation (chitosan ions) and then deacetylation to get the chitosan.

**Fig. 2:**
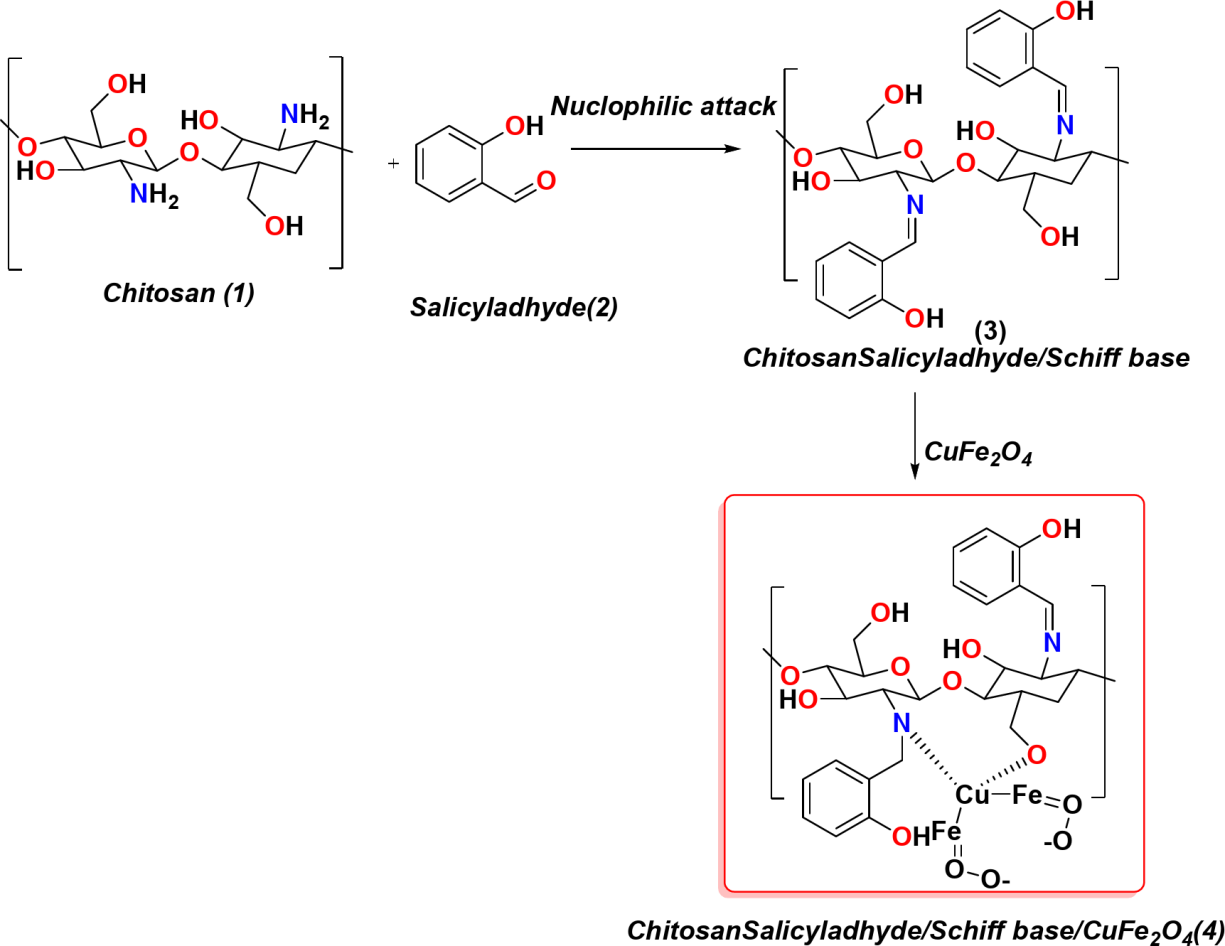
Preparation of Schiff base of novel chitosan/salicylaldehyde and the its bimetallic oxide of CuFe_2_O_4_

Moreover, the reactivity of this chitosan with salicylaldehyde(2) undergoes nucleophilic attack and of the C = O group of aldehyde on the lone pair of the amino group to afford the corresponding chitosan salicylaldehyde/Schiff base(3) which can be chelated easily with CuFe_2_O_4_ bimetallic particles in refluxed acetic acid and chelated the Cu ions with the OH group and NH = of the Schiff base to afford the corresponding chitosan salicylaldehyde/CuFe_2_O_4_ and all compounds were investigated through spectral analysis (Fig. 2).

### FT-IR investigation and NMR of Schiff base

**Fig. 3:**
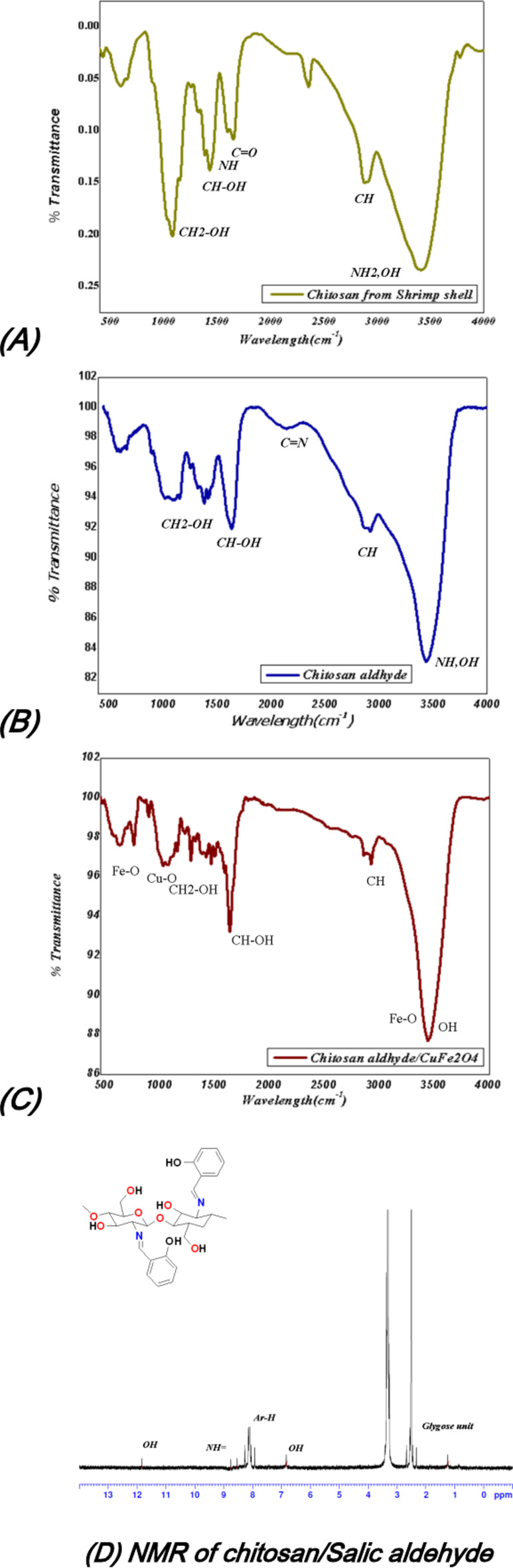
FT-IR analysis of chitosan, chitosan/salicylaldehyde, and chitosan/salicylaldehyde/CuFe_2_O_4_ and (D) 1 HNMR of chitosan/salicylaldehyde

The FT-IR investigation of chitosan, chitosan/salicylaldehyde, and chitosan/salicylaldehyde/CuFe_2_O_4_ showed different absorption bands. Firstly, the chitosan obtained from shrimp showed the presence of NH_2_ at 3400 cm^−1,^ and the OH (3407 cm^−1^) of chitosan showed in the same range as displayed in Fig. 3(A), and also the presence of CH stretching showed 2910 cm^−1^, C = O(1670 cm^−1^) and its NH bending vibration at showed 1450 cm^−1^ and its CH-OH appeared at 1410 cm^−1^. Moreover, the chitosansalicyladhyde (Schiff base) showed the shielded on of amino group and appeared 3340 cm^−1^ and also seen the lower frequencies of amino group due to formation of NH = of Schiff base at 2850 cm^−1^ for stretching vibration while the bending decreased to 1330 cm^−1^ as demonstrated in Fig. 3(B). Additionally the presence of CuFe2O4 made all deformation of the chitosansalicyladhyde and changed its absorption bands which showed the presence of the OH group of chitosan only at 3340 cm^−1^ and the more shielding of the amino group at 3000 cm^−1^ and CH stretching 2290 cm^−1^ and sharp peak at 1650 cm^−1^ which indicates the existence of a strong hydrogen bond between hydroxyl and NH of Schiff base (Fig. 3(C)). Additionally the ^1^HNMR of the chitosan/salicylaldehyde showed the presence of OH of glycose moiety at δ = 6.9ppm, and the presence of phenyl protons of Salicylaldehyde showed multiple signals at 7.9–8.3ppm and the presence of NH = of Schiff proton appeared at 8.7ppm and the OH of salicylaldehyde showed the signal proton at 11.6ppm as showed in Fig. 3(D).

### SEM analysis

**Fig. 4:**
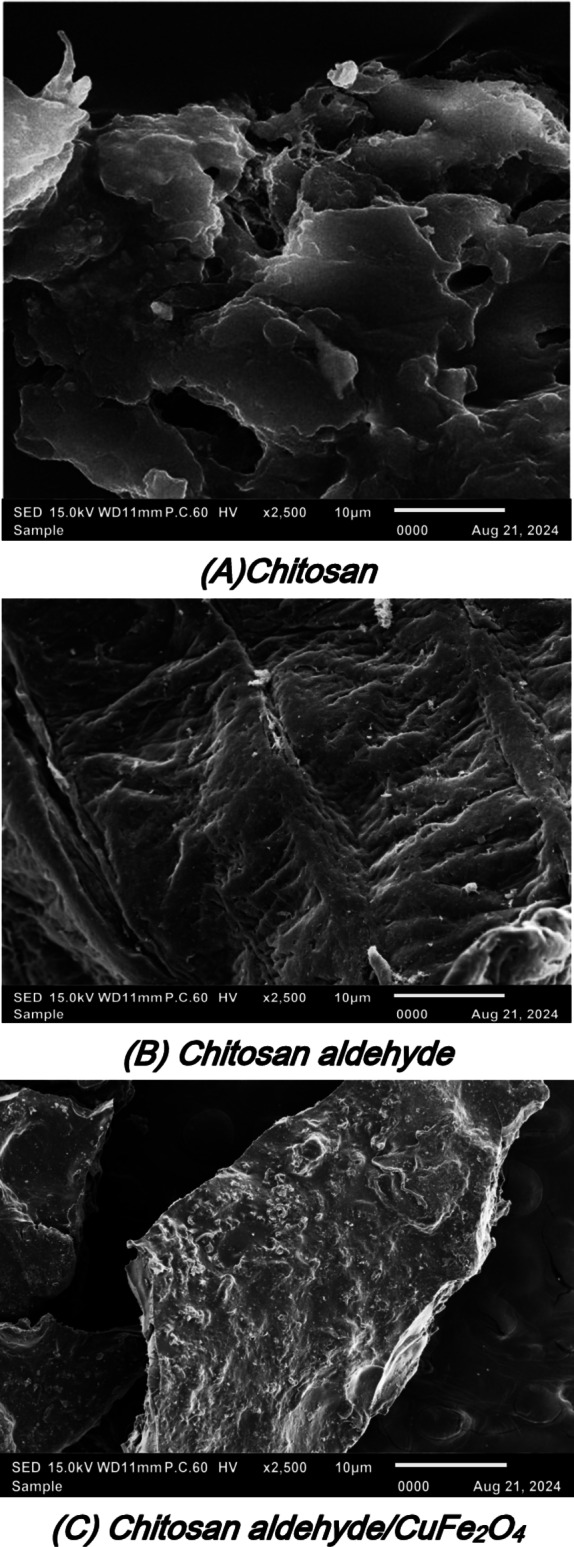
Scanning electron microscope of chitosan, chitosan/salicylaldehyde, and chitosan/salicylaldehyde/CuFe_2_O_4_

The scanning electron microscope was used to investigate the surface of the chitosan, chitosan/salicylaldehyde, and chitosan/salicylaldehyde/CuFe_2_O_4_. Firstly, the morphology surface of chitosan showed a smooth surface and appeared layers above each other and like scratches as displayed in Fig. 4(A). The SEM of the chitosan/salicylaldehyde showed the changing of the surface of chitosan to the more smooth surface which showed small holes on the surface like waves due to the formation of Schiff base (Fig. 4(B))**.** Furthermore, the chitosan/salicylaldehyde/CuFe_2_O_4_ showed the presence of CuFe_2_O_4_ accumulation on the surface of and made the surface a shiny rock and indication of hydrogen bonding interaction of these nanomaterials with chitosan/salicylaldehyde (Fig. 4(C)).

### XRD diffraction

**Fig. 5:**
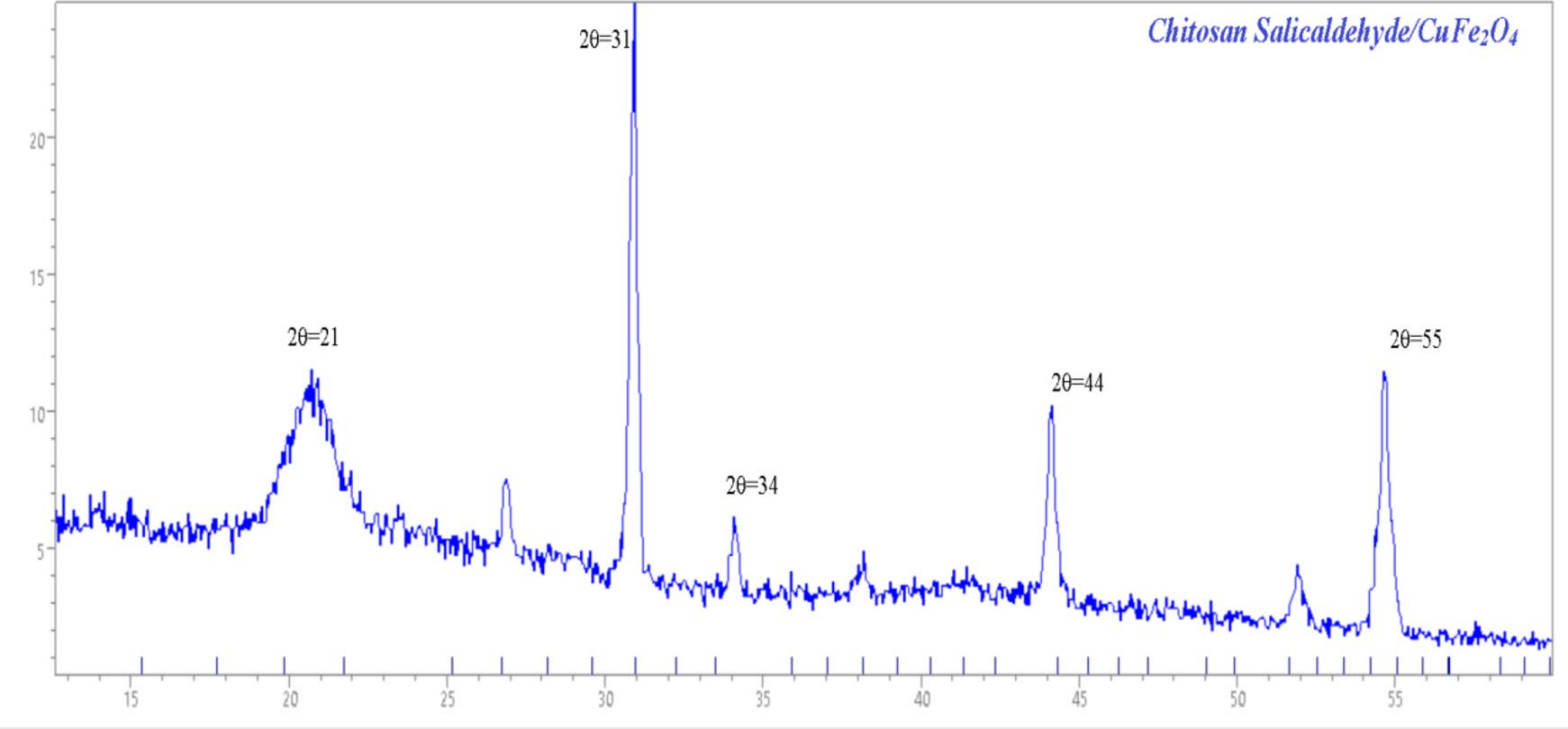
XRD pattern of chitosan/salicylaldehyde/CuFe_2_O_4_

The crystallinity of chitosan salicylaldehyde/CuFe_2_O_4_ was characterized through an X-ray diffraction pattern as displayed in Fig. 5. The XRD patterns for chitosan/salicylaldehyde/CuFe_2_O_4_ obtained from treatment of chitosan Salicaldehyde with CuFe_2_O_4_which showed the different diffraction peaks at 2θ = 21 which indicate for semicrystalline nature of chitosan^23^, and the PXRD profile of CuFe_2_O_4_ showed its characteristic intensity at 2θ = 31, 34, 44, 55 and its showed the presence on the surface of chitosan and changed its morphology as displayed in Fig. 5.

## Biological investigation

### ADME studies

A crucial initial phase in the drug development process is computer analysis, which provides valuable data that directs the subsequent biological analyses. Moreover, generated heterocyclic compounds’ characteristics and behaviors are examined using computer methods. These medications’ physicochemical properties have a significant impact on their pharmacodynamics and pharmacokinetics^39–42^. 

**Fig. 6:**
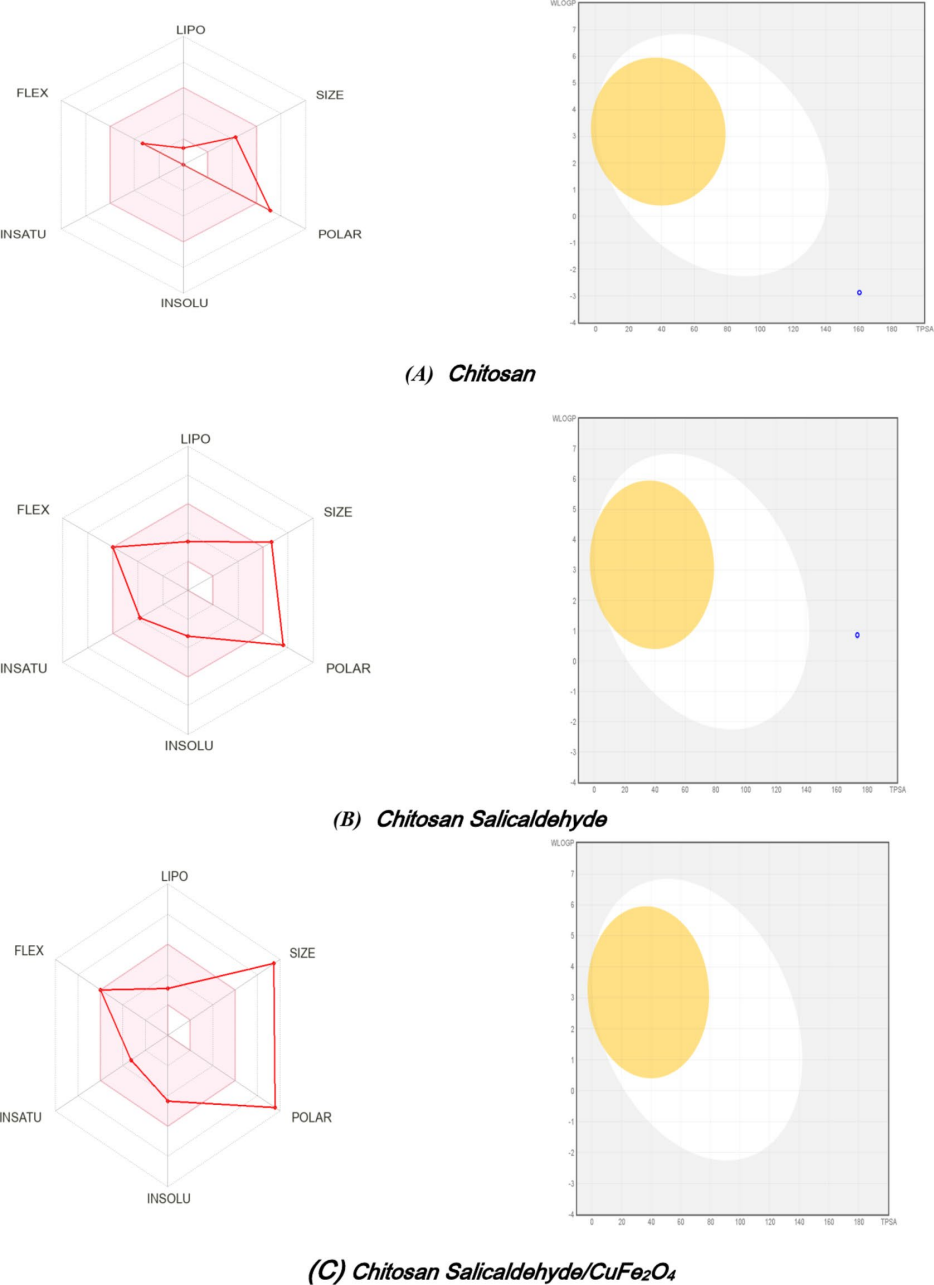
The ability to be bioavailable The physicochemical characteristics radar and egg ball (Swiss ADME analysis)

The effective development of pharmaceuticals and other industrial chemicals depends on a detailed understanding of a molecule’s absorption, distribution, metabolism, excretion, and toxicity (ADME) properties^41,43^. The Swiss ADME web server and the PubChem database’s physicochemical metrics were utilized to assess the phytochemicals’ drug-likeness. We evaluated the drug-like properties of phytochemicals derived from heterocyclic compounds using Lipinski’s Rule of Five. In light of Fig. 6; Table 2, the molecular weight of chitosan showed C_15_H_30_N_2_O_7_ which has 6 hydrogen donors and 9 hydrogen acceptors, suggesting stability and potential for significant interactions. Chitosan demonstrated a high Topological Polar Surface Area (TPSA) of 160.65Å^2^, showing the ability to penetrate drug transport characteristics, and a high lipophilicity (log *P* = 2.29), indicating a tendency to dissolve in lipids. As per the bioavailability reader, the pink region denotes the optimal range for every attribute, which includes polarity (log S not exceeding 0.45 but soluble), size (MW is 350.41 g/mol), and lipophilicity (XLOGP3 is −3.25). The molar refractivity is equivalent to the molar volume adjusted by the refractive index. It displays the size and polarizability of a fragment or molecule. It demonstrated the high concentration of CuFe_2_O_4_(153.19), and the compound’s polar surface displayed 227.94 Å^2^, greater than that of chitosan salicylaldehyde (173.79 Å²) and the rise in donor proton. The boiled eggs absorbed the gastrointestinal tract and these results directed an examine these compounds in anti-tumor activities^44–46^.

**Table 2:** Physicochemical characteristics of derivatives of synthetic chitosan

Compound number	Molecular weight (g/mol)	Lipophilicity (log *p*)	Hydrogen bond donors	Hydrogen bond acceptors	TPSA (Å^2^)	Log S	Molar Refractivity
***Chitosan***	C_15_H_30_N_2_O_7_	2.29	6	9	160.65Å^2^	0.45	83.31
***Chitosan salicylaldehyde***	C_29_H_38_N_2_O_9_	3.13	6	11	173.79	−3.62	147.90
***Chitosan salicylaldehyde/CuFe2O4***	C_29_H_42_CuFe_2_N_2_O_13_	0.00	8	15	227.94	−2.08	153.19

### Assessment of in vitro cytotoxic action

**Fig. 7:**
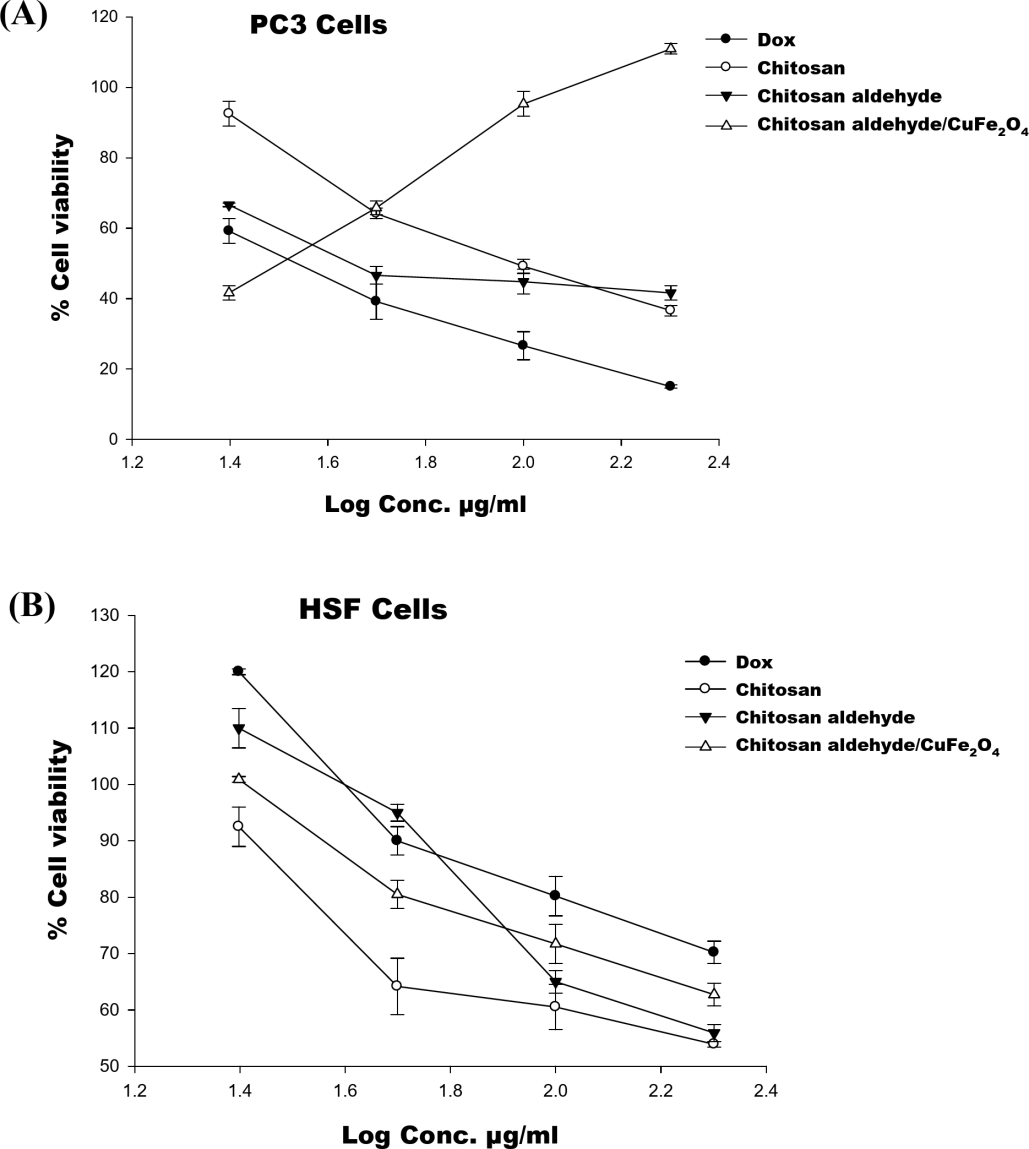
Effects of the investigated chitosan compounds on **PC3** and **HSF** cells were assessed at 48 h

The cytotoxicity action of chitosan, chitosan salicylaldehyde, and chitosan salicylaldehyde/CuFe_2_O_4_ against **PC3** and **HSF** cell lines at variable concentrations (25, 50, 100, and 200 µg/ml) was assessed utilizing the neutral red test, which is dependent on the ability of viable cells to bind and integrate the supravital dye neutral red into their lysosomes. When compared to untreated cells, doxorubicin (Dox), the reference drug, had a significant impact on **PC3** cell development after 48 h. The use of DMSO as a solvent had no noticeable influence on the growth of **PC3** and **HSF** cells upon a 48-hour treatment. The percentage of cell viability obtained from the neutral red test is displayed in Fig. 7**(A**,** B)**^47^. Chitosan salicylaldehyde/CuFe_2_O_4_ demonstrated a significant inhibitory effect, with an IC_50_ of 35.3 µg/ml, relative to Dox (IC_50_ = 39.19 µg/ml) (*P* < 0.05). Also, chitosan salicylaldehyde (IC_50_ = 45.55 µg/ml) exhibited a more inhibitory impact than the starting compound, chitosan (IC_50_ = 96.78 µg/ml), when treated with **PC3** cells. Furthermore, chitosan and its derivatives exhibit no cytotoxicity when tested against the **HSF** human normal skin fibroblast cells, demonstrating the safety of these compounds. These findings showed that chitosan salicylaldehyde and chitosan salicylaldehyde/CuFe_2_O_4_ had a greater inhibitory effect on the growth of **PC3** cells.

### SAR

Structure-activity relationship (SAR) studies demonstrated how the combination of salicylaldehyde with chitosan and contributions of their differences in the cytotoxic effect of chitosan. The presence of the salicylaldehyde/CuFe_2_O_4_ moiety in chitosan salicylaldehyde/CuFe_2_O_4_, followed by the salicylaldehyde moiety in chitosan salicylaldehyde, is more cytotoxic than chitosan when treated with **PC3** cells for 48 hour. These results showed the importance of the salicylaldehyde and CuFeo_3_ as a pharmacophores for the anticancer effect.

### Docking investigation of PC3 cells

**Fig. 8:**
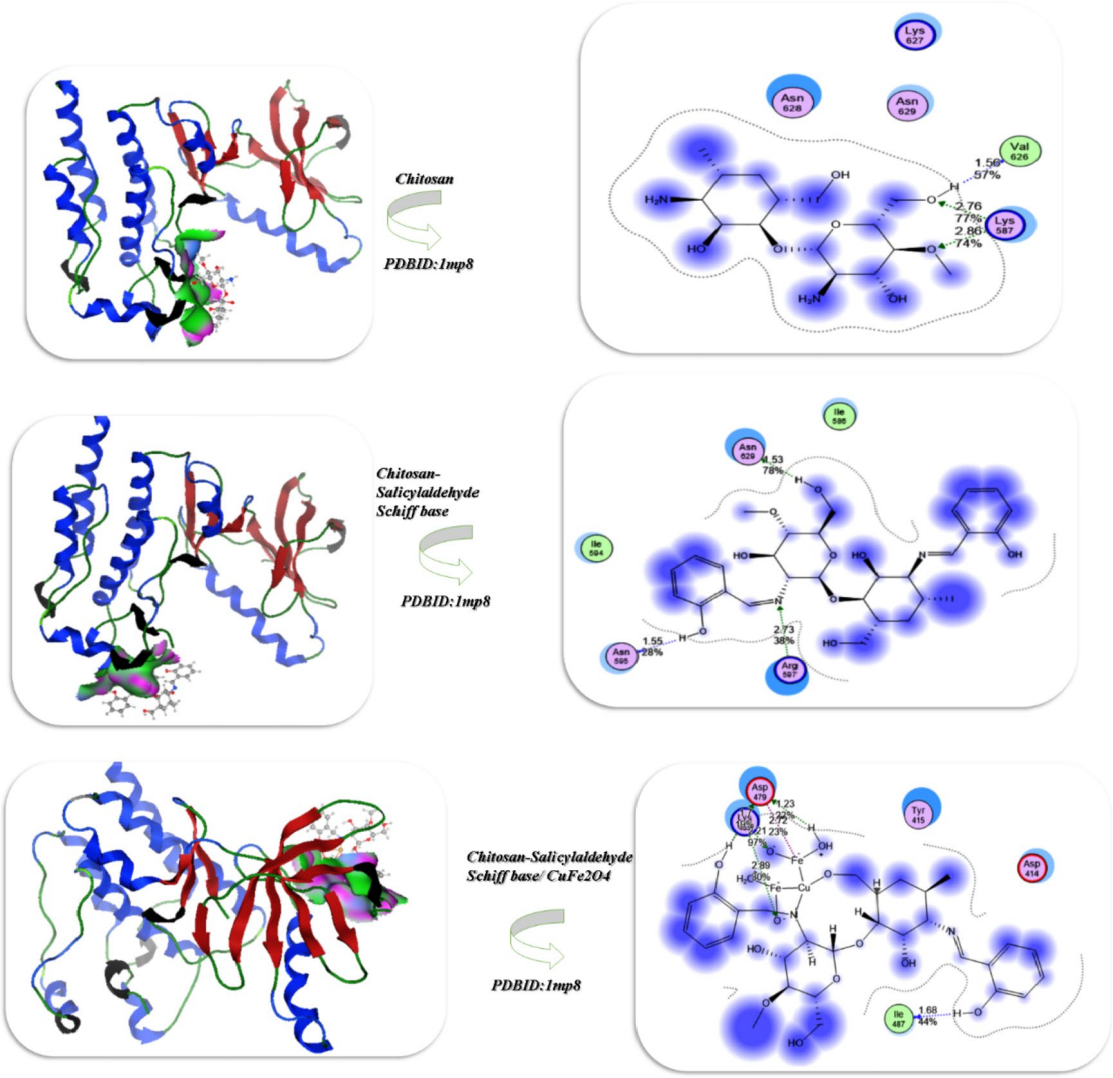
Results of the docking investigation of the chitosan derivatives with PDBID :1mp8.

We conducted the docking simulation of chitosan, chitosan salicylaldehyde, and chitosan salicylaldehyde/CuFe_2_O_4 _utilized flexible docking rotation inside the pocket of protein. The simulation was performed within the Crystal structure of Focal Adhesion Kinase (FAK)(PDBID:1mp8)^33^. The docking analysis of chitosan with **PDBID**:1mp8 showed 9.34 kcal/mol for its binding affinity and its bond length showed 1.56, 2.76Å and bonded with different amino acids such as Val 626, Lys 587, Lys 627, Asn 629, Asn 628, while the increasing of interaction between chitosan salicylaldehyde and the PDBID:1mp8 showed binding affinity with 12.87 kcal/mol and interact the amino acids such as Arg597, Asn629, Asn 595, Ile 586 which interact with NH = of imine linkage which interact easily with shortage length 1.55, 2.73, 1.53Å, moreover, the presence of CuFe_2_O_4_ and their chelation with chitosan salicylaldehyde which gave its ability of interaction with Fe = O bond with total binding energy 16.98 kcal/mol and its length 1.23,2.89, 1.68, 2.72Å and contacted with Asp 479, Lys 433, Tyr 415, Asp 414, Ile 487 as displayed in Fig. 8; Table 3.

**Table 3:** Docking simulation of chitosan, chitosan aldehyde, and chitosan aldehyde/CuFe_2_O_4_ with **PDBID**:1mp8.

PDBID:1mp8			
	Energy affinity(kcal/mol)	Distance(Å)	Amino acids
***Chitosan(1)***	9.34	1.56, 2.76	Val 626, Lys 587, Lys 627, Asn 629, Asn 628
***Chitosan salicylaldehyde(3)***	12.87	1.55, 2.73, 1.53	Arg597, Asn629, Asn 595, Ile 586
***Chitosan salicylaldehyde/CuFe***_***2***_ ***O***_***4***_ ***(4)***	16.98	1.23,2.89, 1.68, 2.72	Asp 479, Lys 433, Tyr 415, Asp 414, Ile 487

### Gene expression outcomes

The effects of chitosan, chitosan salicylaldehyde, and chitosan salicylaldehyde/CuFe_2_O_4_ on **PC3** cells treated with them were evaluated using the IC_50_ values of these compounds after 48 h. The mRNA expression levels of PI3K, AKT, mTOR, and CCND1 were measured by calculating the ratio of their expression to that of β-Actin and comparing the results to control values. Previous research has shown that **PC3 **cells had up-regulated expression levels of PI3K, AKT, mTOR, and CCND1^48–52^.

Prostate cancer is still the most frequently diagnosed noncutaneous cancer^53,54^. Although there is a nearly 100% survival rate for individuals with assumed localized prostate cancer, patients may have a recurrence of the illness years after the prostate is removed, indicating that PC cell dispersion begins early in tumor growth^55^. Many investigations have shown that prostate cancer is significantly influenced by the PI3K-AKT-mTOR pathway^56^. This signaling pathway regulates cell migration, proliferation, survival, and differentiation and is linked to autophagy^57,58^. Akt is the principal mediator of PI3K signaling, with mTOR as a downstream target of this PI3K/Akt signalling pathway^59,60^. The activity of two distinct complexes (mTORC1 and mTORC2) largely controls the functioning of mTOR; in particular, the kinase activity of mTORC1 suppresses autophagy and promotes homeostatic cell growth, proliferation, and survival. It has been demonstrated that PI3K-Akt-mTOR activity suppresses autophagy while blocking this activity increases autophagic activity.

Additionally, mTOR phosphorylation at Ser2448 allows activated AKT to directly activate mTORC1. When TSC2 is phosphorylated by AKT, TSC1 and TSC2 are reduced, which activates mTORC1. Rheb-GTP increased mTORC1 activity in response to AKT-induced TSC2 inactivation, which in turn phosphorylated 70S6K1, S6, and eukaryotic translation initiation factor 4Ebinding protein 1 (4EBP1)^61^. Also, 4EBP-1 phosphorylation stimulates the translation of mRNA encoding cyclin D1, c-Myc, and hypoxia-inducible factor 1α, which results in angiogenesis or the development of the cell cycle^62^. Increased cyclin D1 mRNA expression, splice variations, transcript aberrations, and downregulated cyclin D1 degradation are the usual causes of a high level of cyclin D1^63,64^. Among these, dysregulated cyclin D1 degradation may be a major contributing factor. When double-strand DNA breaks (DSBs) occur, the inability to degrade cyclin D1 can result in the accumulation of DNA damage, which can accelerate the development of cancer^38,39^. Moreover, the inability to degrade cyclin D1 leads to a compromised intra-S-phase checkpoint and elevated cyclin D1 expression, which is frequently observed in several types of malignant tumors and is indicative of a bad prognosis^65^.

**Fig. 9:**
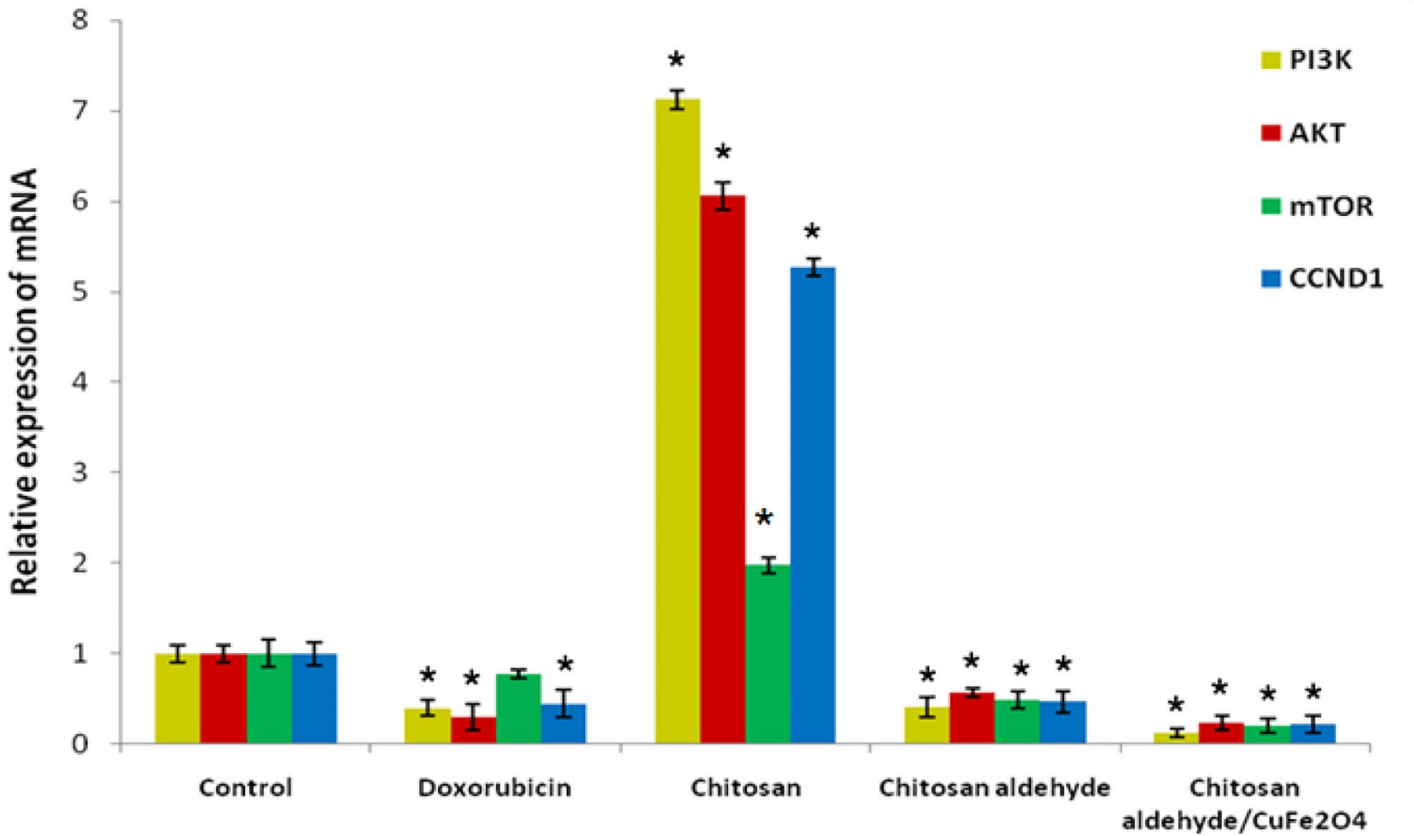
Impacts of doxorubicin and the studied chitosan compounds on PI3K, AKT, mTOR, and CCND1 gene mRNA expression in **PC3** cells. Each result displays the mean ± SE from three separate studies, *P < 0.05 relative to the control.

Results suggested that doxorubicin significantly down-regulated the expression levels of PI3K, AKT, and CCND1, whereas it was non-significantly down-regulated in **PC3** cells compared to untreated cells (*P* < 0.05) (Fig. 9). On the other hand, gene expression levels of PI3K, AKT, mTOR, and CCND1 were significantly decreased by treating **PC3** cells with chitosan salicylaldehyde/CuFe_2_O_4_ and chitosan salicylaldehyde, while chitosan did not affect these genes in **PC3** cells at 48 h compared to untreated cells (*P* < 0.05) (Fig. 9). Also, the figure shows the amplification plot and the melting curve for the studied genes is presented in the supplementary data file: Fig. S1. According to these results, chitosan salicylaldehyde/CuFe_2_O_4_ may have a greater impact than chitosan salicylaldehyde in reducing the expression of PI3K, AKT, mTOR, and Cyclin D1, which in turn prevents the survival and proliferation of **PC3 **prostate cancer cells^66^.

### Docking of gene expression

**Fig. 10:**
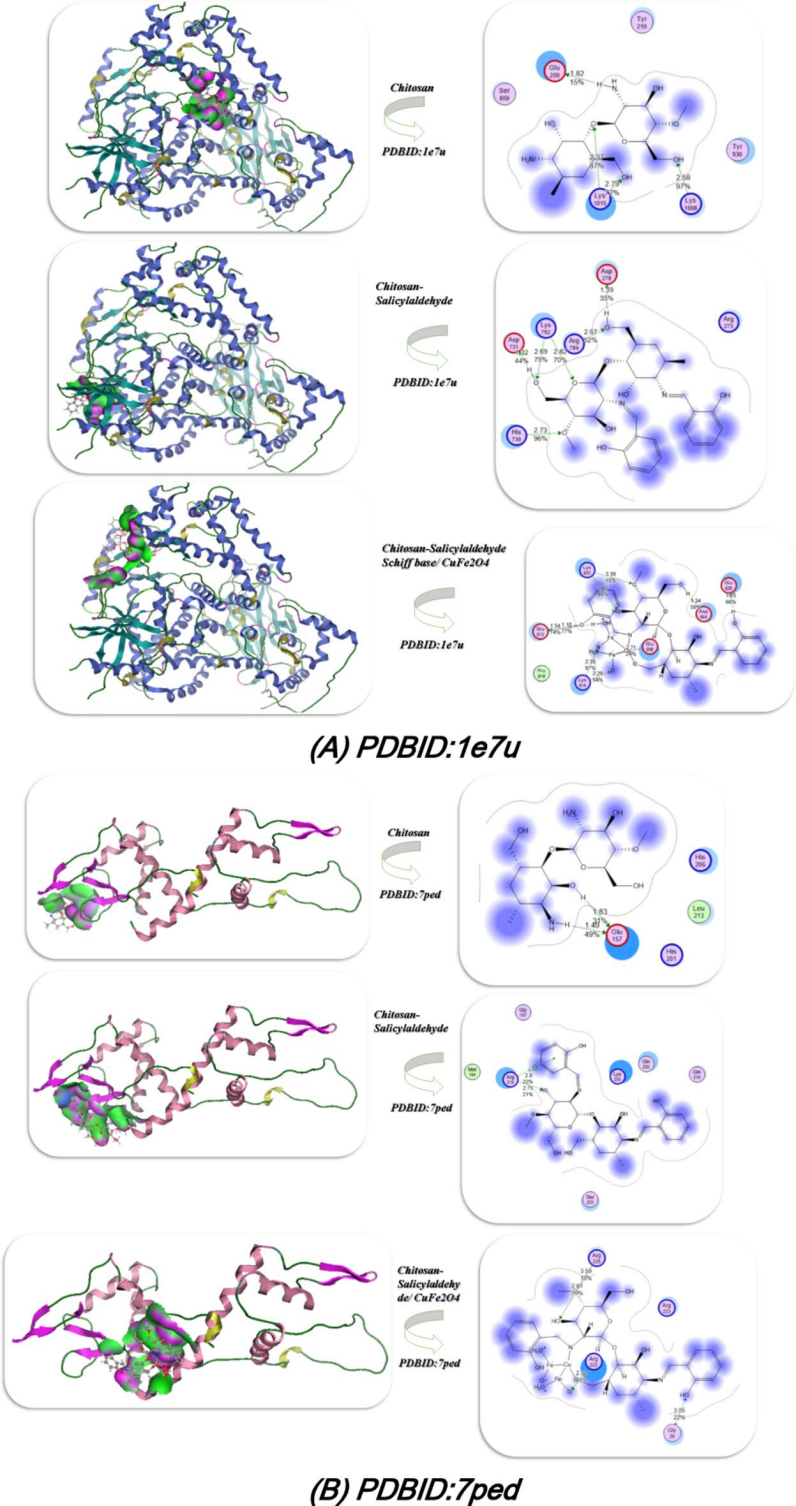

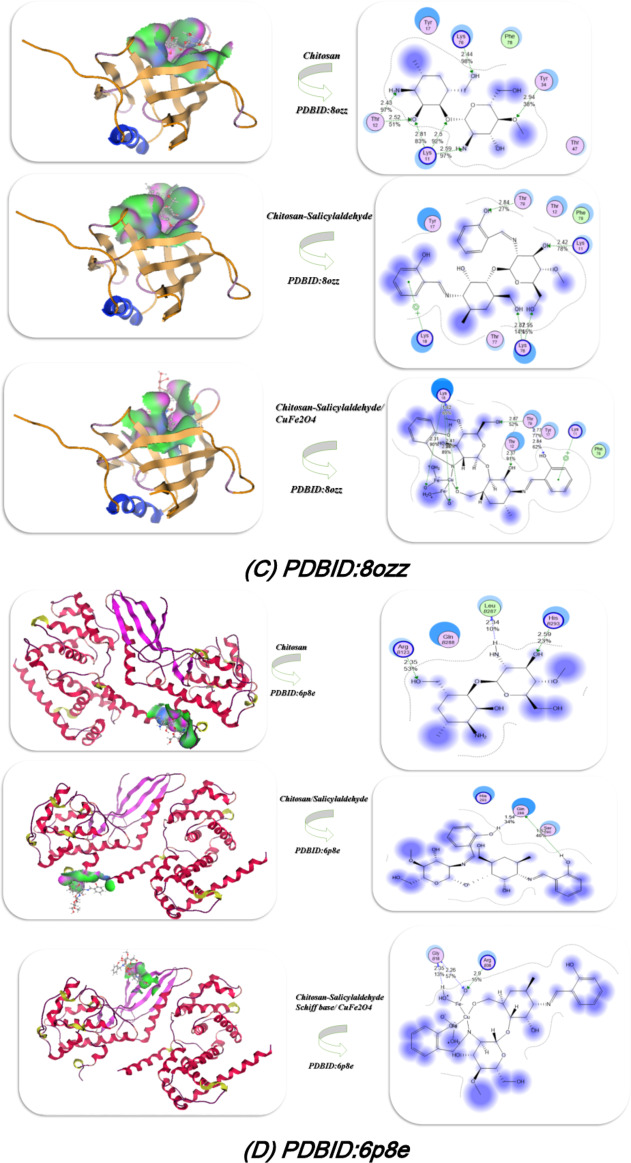
Docking investigation of the chitosan derivatives with PDBID : 1e7u, 7ped, 8ozz and PDBID : 6p8e

Docking analysis of gene expression which be characterized through different proteins such as PH domain of AKT-like kinase in Trypanosoma cruzi(**PDBID**:8ozz)^34^, DEPTOR DEP domain tandem (DEPt)(**PDBID**:7ped)^35^, Crystal structure of CDK4 in complex with CyclinD1 and P27(**PDBID**: 6p8e)^36^, Structure determinants of phosphoinositide 3-kinase inhibition by wortmannin, LY294002, quercetin, myricetin and staurosporine (**PDBID**:1e7u)^37^ which is demonstrated at Fig. 10; Table 4. The docking interaction of chitosan derivatives with **PDBID**:1e7u which showed the most binding with chitosan salyicaldehyde/CuFe_2_O_4_(4) with total binding energy − 11.98 kcal/mol and its length distance 1.45, 0.35, 3.39, 1.34, 1.25, 2.71Å and their amino acids Lys 920, Glu 913, Glu 956, Glu 826, Asp 884, Glu 956 and chelated with C = O and the OH of chitosan, and also the Schiff base showed excellent interaction with this protein also − 9.65 kcal/mol and distance 1.39, 2.69, 2.82, 2.73Å, and interact with the imine linkage also and the chitosan showed least binding with this protein with − 6.5 of its total energy and mostly interact with amino and hydroxyl group. Furthermore, the docking analysis of **PDBID**: 7ped which also showed the tendency of the presence of CuFe_2_O_4_ with chitosan to increase its action and interact inside the pocket of protein with − 12.45 kcal/mol and attached with distance 3.59, 2.32, 3.05Å and its amino acids Arg 225, Arg 223, Gly 20, and its Schiff base also showed high activity inside the protein pocket with − 11.98 kcal/mol and involved with 2.8, 2.75Å (Arg 218, Lys205, Gln 200, Gln 216). Additionally the interaction with the PDBID:8ozz and 6p8e showed the chitosan/salicylaldehyde/CuFe_2_O_4_ most bonded then the chitosan/salicylaldehyde and its correlated with the experimental section which indicated that the presence of NH = in chitosan/salicylaldehyde enhanced the activity and its addition of CuFe_2_O_4_ nanomaterials and its interaction inside the chitosan/salicylaldehyde gave chance for formation more electrostatic hydrogen bond interaction between the protein’s and the compound and increase its action.

**Table 4:** Molecular docking outcomes of chitosan, chitosan/salicylaldehyde, and chitosan/salicylaldehyde/CuFe_2_O_4_ with different proteins

PDB ID:6p8e					PDBID: 1e7u		
	*Energy affinity(kcal/mol)*	*Distance(Å)*	*Amino acids*		*Energy affinity (kcal/m* *ol)*	*Distance(Å)*	*Amino acids*
***Chitosan(1)***	−8.65	2.35, 2.34, 2.59	Arg B123, Leu B287, His B293	***Chitosan(1)***	−6.54	1.82, 2.37, 2.58,2.79	Glu 209, Tyr 210, Lys 108, Tyr 930,
***Chitosan salicylaldehyde(3)***	−10.65	1.54, 1.52	His 293, Gln 288, Ser 290	***Chitosan salicylaldehyde(3)***	−9.65	1.39, 2.69, 2.82, 2.73	His 730, Lys 792, Arg 784, Asp 278, Asp 731
***Chitosan salicylaldehyde/CuFe***_***2***_ ***O***_***4***_ ***(4)***	−14.87	2.9, 2.35, 2.26	Arg B38, Gly B18	***Chitosan salicylaldehyde/CuFe***_***2***_ ***O***_***4***_ ***(4)***	−11.98	1.45, 0.35, 3.39, 1.34, 1.25, 2.71	Lys 920, Glu 913, Glu 956, Glu 826, Asp 884, Glu 956
***PDBID:8ozz***				***PDBID:7ped***			
***Chitosan(1)***	−10.12	2.44, 2.94, 2.5, 2.81, 2.43	Lys 76, Thr 12, Lys 11, Tyr 34,	***Chitosan(1)***	−10.76	1.83, 1.49	Glu 157, His 201, Leu 213, His 206
***Chitosan salicylaldehyde(3)***	−10.67	2.42, 2.82, 2.84	Thr 79, Thr 12, Phe 78, Lys 11, Lys 76	***Chitosan salicylaldehyde(3)***	−11.98	2.8, 2.75	Arg 218, Lys205, Gln 200, Gln 216
***Chitosan salicylaldehyde/CuFe***_***2***_ ***O***_***4***_ ***(4)***	−11.87	2.77, 2.31, 2.87	Lys 18, Thr 79, Tyr 17, Lys 11, Phe 78,	***Chitosan salicylaldehyde/CuFe***_***2***_ ***O***_***4***_ ***(4)***	−12.45	3.59, 2.32, 3.05	Arg 225, Arg 223, Gly 20,

## DFT investigation

### Optimization of Chitosan derivatives

**Fig. 11:**
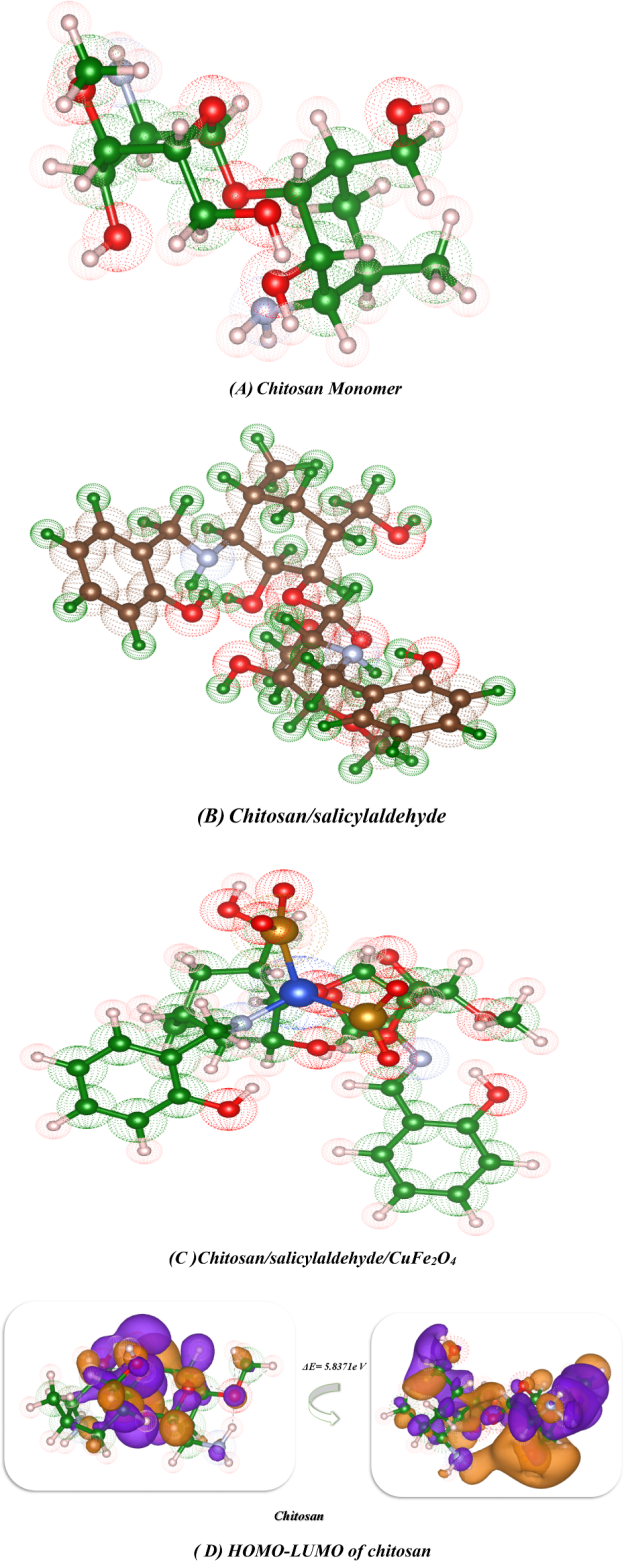

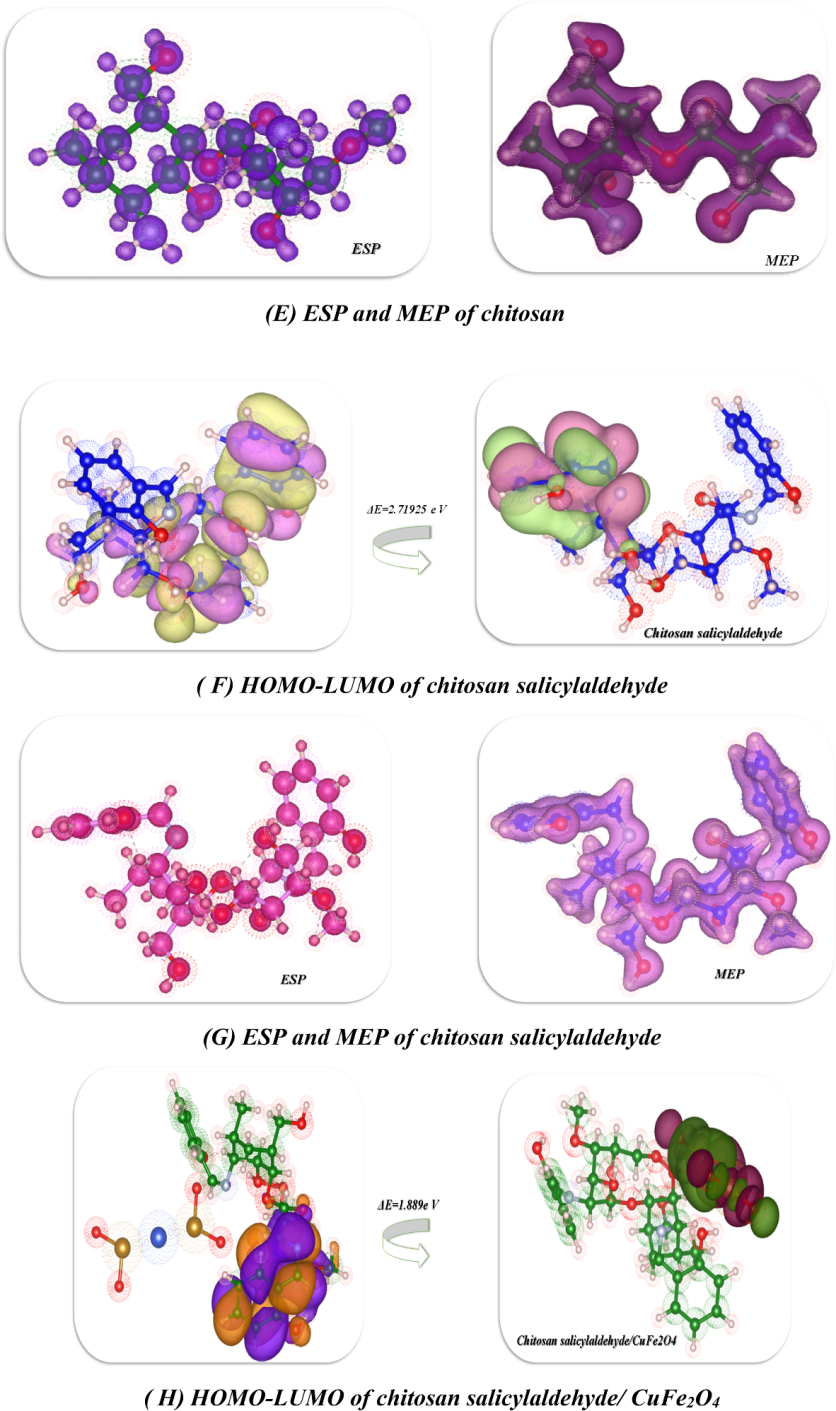

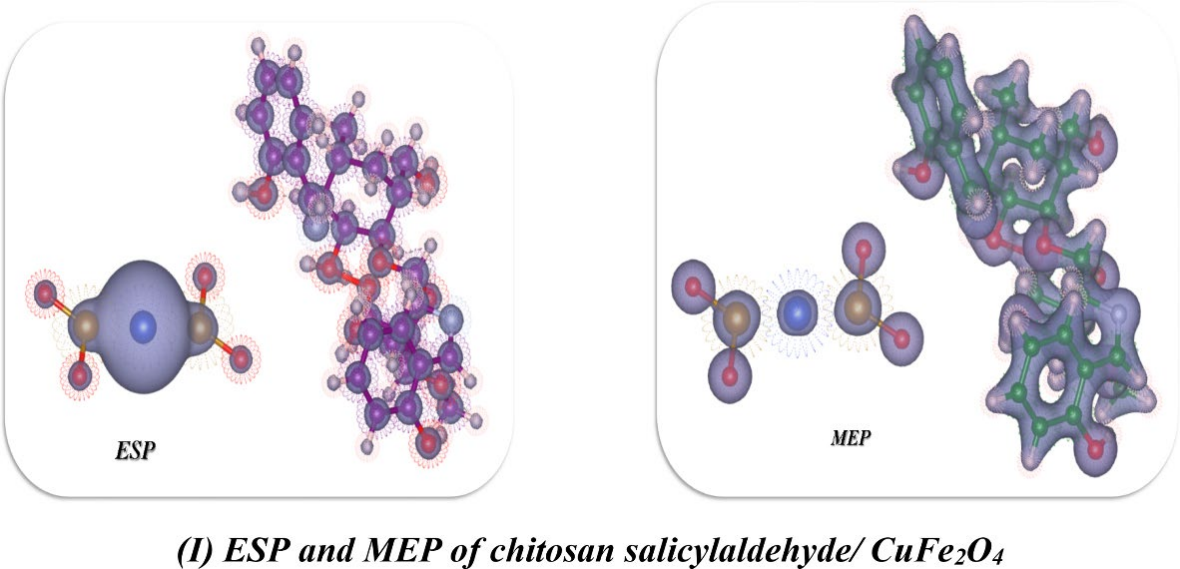
(**A**-**I**) Optimized structure of compounds chitosan, chitosan salicylaldehyde, and chitosan salicylaldehyde /CuFe_2_O_4_ utilizing DFT/B3LYP/LAN2DZ(G) basis set.

In this study, we optimized the chitosan derivatives utilized Gaussian(09)^67,68^ through DFT/B3LYP/LAN2DZ(G) basis set. Moreover, the physical characteristics used in the optimization of molecular structures of chitosan, chitosan calicylaldehyde and chitosan Salicylaldehyde CuFe_2_O_4 _were concerning (σ) absolute softness^69^, (χ) electronegativities^70^, (ΔN_max_) electronic charge^71^, (η) absolute hardness, (ω)^72^global electrophilicity^73^, (S) global softness^74^, and (Pi) chemical potential^75^, from the Eqs^1–8^which were scheduled in Table 5; Fig. 11^42,46,76–78^.

**Table:** 

$\Delta E=E_{LUMO}-E_{HOMO}$	***[1]***	$\chi=\frac{-(E_{HOMO}+E_{LUMO})}{2}$	***[2]***
$\eta=\frac{(E_{LUMO}-E_{HOMO})}{2}$	***[3]***	$\sigma=1/\eta$	***[4]***
$Pi=-\chi$	***[5]***	$S=1/2\eta$	***[6]***
$\omega=Pi^2/2$	***[7]***	$\Delta N max=-Pi/\eta$	***[8]***

The DFT analysis of the chitosan derivatives revealed that every compound was non-planar, as seen in Fig. 11. Table 4 provides the physical characteristics of each compound. First, the optimization of the chitosan yielded a total energy of (−33335.8497 e V) (−768742.7625 kcal/mol), indicating the stability of this compound. Additionally, the gap band energy between the HOMO and LUMO was found to be 5.837 e V, a high value that results from electron delocalization around the NH_2_ and OH groups, increasing the electrostatic hydrogen bonding interaction, as shown in Fig. 11(A) and its dipole moment showed 6.4147 Debye which is highly electronegative and can easily attract bond pair electrons and react again, the atomic affinity for interacting with a pair of electrons is represented by the absolute electronegativity (χ), and it showed 1.551e V which has less electronegativity, while its chemical hardness (η), reflecting the resistance to changes in electron cloud density within the system, exhibited a 2.919e V due to the influence of nitrogen atoms of amino group attributed with hydroxy groups of glycoses ring which increase its hardness. Furthermore, the chitosan salicylaldehyde showed total energy − 52066.143905 e V which is less in its energy than the chitosan due to formation of Schiff base and its linkage of imine which gave the stability of compound and its showed the more electron transfer between HOMO-LUMO stages with 2.719 e V which indication of activity for Schiff base and its dipole moment showed 9.5053Debye and which indicate its can easily charge separation and its electronegativity showed 2.827e V which indication of its ability to interact it again and its hardness also reduced to 1.360e V that showed the roughness of surface due to formation of NH=, also in presence of chelation of CuFe_2_O_4_ with the chitosan salicylaldehyde to afford the chitosan salicylaldehyde/CuFe_2_O_4_which gave the stability of compound and its total energy (−61400.649699e V)^79,80^and due to electrostatic hydrogen bond interaction between these nanomaterials oxide with chitosan salicylaldehyde gave the gap energy between HOMO-LUMO showed (1.889 e V) and its reduce the hardness of the surface of chitosan to gave its less value than chitosan aldehyde to 0.944 e V and these results for formation of more nitrogen atoms inside the chitosan ring with the Cu ions which increase the softness to it to 0.529 e V while which is in the chitosan salicylaldehyde (0.368e V), Also, ESP and MEP are methods used to study and forecast molecular behavior and were also utilized to identify the electrophilic and nucleophilic active sites as well as hydrogen bonding interactions^46,52,81^. It provides a visual representation of the charge distribution in three dimensions and helps comprehend the relative polarity of a molecule. This is an extremely useful tool for understanding both the physicochemical qualities and molecular structure. Using DFT/B3LYP/LAN2DZ(G) basis set optimized geometry, molecular electrostatic potentials (MEP) were calculated to predict active sites for electrophilic and nucleophile reactions inside the molecule, as shown in Fig. 11**(A-I). **Chitosan derivative’s positive charges and negative areas were discovered to be dispersed inside the pocket and most delocalization on the surface of bimetallic oxide which enhanced biological activities, supporting its biological assessment^31,79,82^.

**Table 5:** The physical descriptors for compounds chitosan, chitosan/salicylaldehyde, and chitosan/salicylaldehyde/CuFe_2_O_4_ utilizing the DFT/B3LYP/LAN2DZ(G) basis set

	Chitosan	Chitosan/ Salicylaldehyde	Chitosan/ Salicylaldehyde/CuFe_2_O_4_
***E***_***T***_ ***(au)***	−1225.06931	−1913.3946	−2256.4312
***E***_***HOMO***_ ***(eV)***	−4.46977	−4.18677	−2.9876
***E***_***LUMO***_ ***(e v)***	1.36738	−1.46752	−1.09876
***E***_***g***_ ***(eV)***	5.837	2.719	1.889
***µ (D)***	6.4147	9.5053	14.8711
***χ (eV)***	1.551	2.827	2.043
***η(eV)***	2.919	1.360	0.944
***σ(eV)***	0.343	0.735	1.059
***Pi(eV)***	−1.551	−2.827	−2.043
***S(eV)***	0.171	0.368	0.529
***ω(eV)***	0.412	2.939	2.210
***ΔN max***	0.531	2.079	2.163

### Conclusion

In these studies, we showed the interaction of bimetallic oxide of CuFe_2_O_4_ on the surface of chitosan salicylaldehyde which exhibited the framework for the suppression of **PC3** cell proliferation in response to chitosan salicylaldehyde/CuFe_2_O_4_ by reduced expression levels of CCND1, and mainly by inhibition of the PI3K/AKT/mTOR signalling axis. All of these contribute to the inhibition of the proliferation, survival, differentiation, and induction of autophagy in prostate cancer cells, and chitosan salicylaldehyde/CuFe_2_O_4_ might potentially serve as a potential candidate for cancer therapy. These biological results were verified by docking investigation and DFT/B3LYP/LAN2DZ(G) basis set, which showed a good correlation with experimental analysis.

## Electronic supplementary material

Below is the link to the electronic supplementary material.

Supplementary Material 1

## Data Availability

All data generated or analyzed during this study are included in this Article.
