# Supplementary material for: Studying the impact of chitosan salicylaldehyde/schiff base/CuFe2O4 in PC3 cells via theoretical studies and inhibition of PI3K/AKT/mTOR signalling
Source: Sci Rep. 2025 Feb 3;15:4129. doi: 10.1038/s41598-025-86096-7 (PMC11790862; doi:10.1038/s41598-025-86096-7)
Supplement: Supplementary file 1 — Supplementary Material 1 [file 41598_2025_86096_MOESM1_ESM.doc]

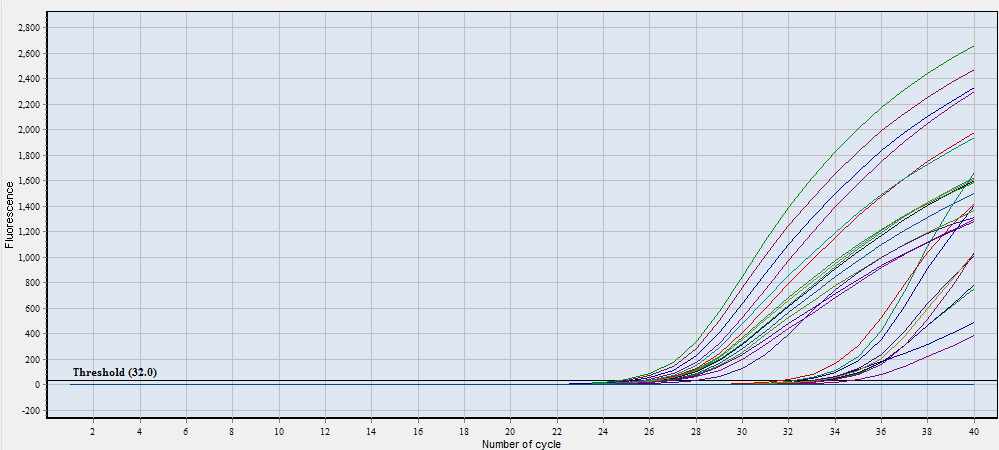


**(A)**


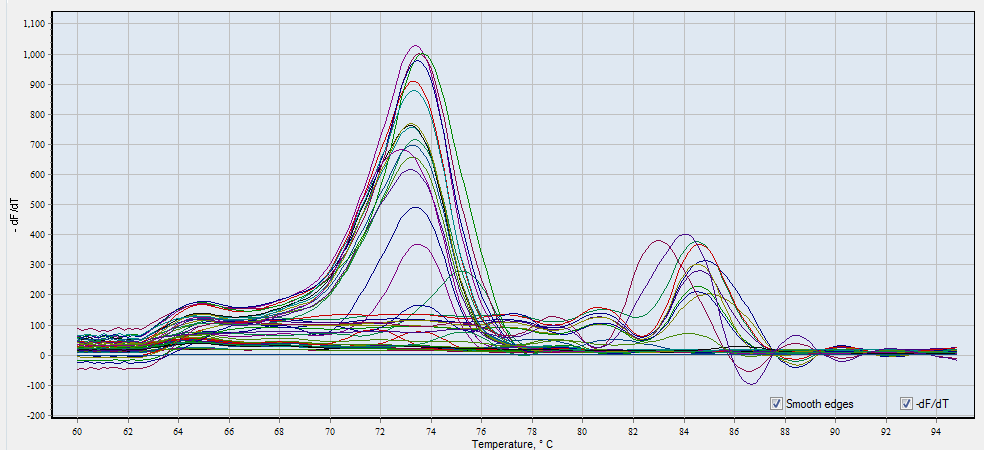


**(B)**


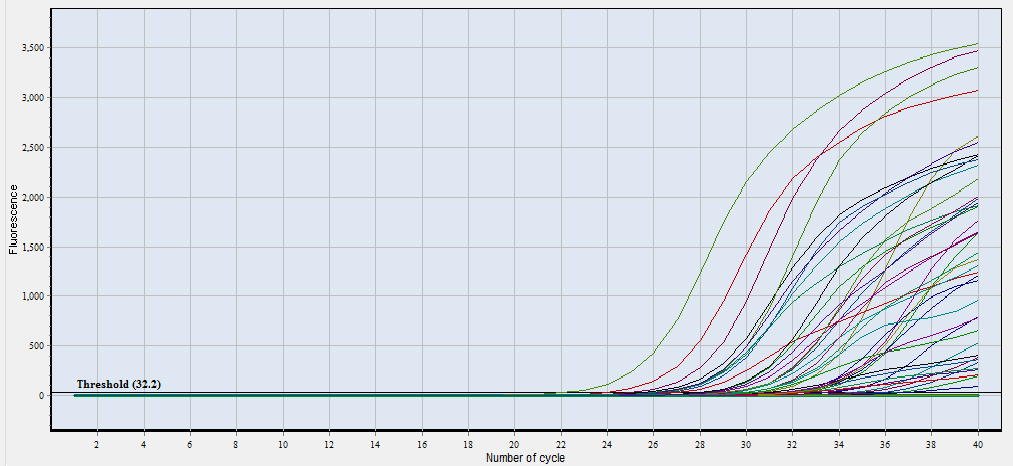


**(C)**


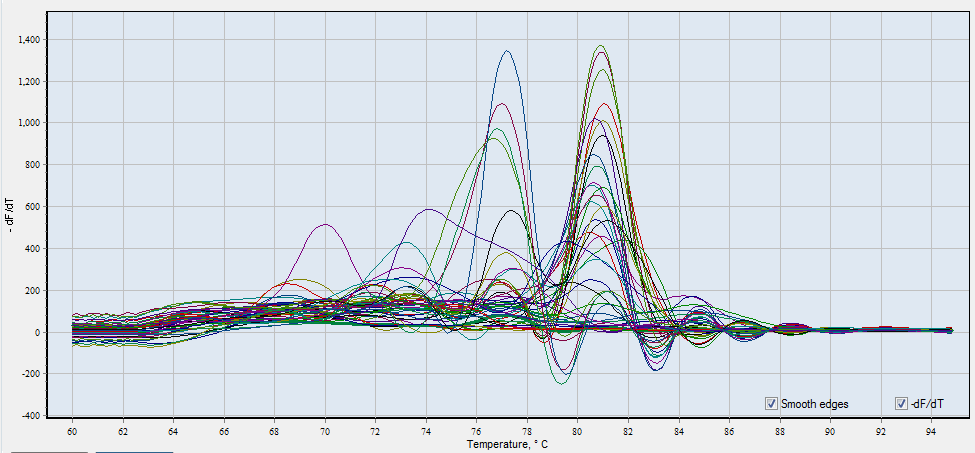


**(D)**

**Fig. 10:** the amplification plot and the melting curve for the studied genes. **A, B**: For β-actin and CCND1 genes. **C, D**: For PI3K, AKT, and mTOR genes.
